# Supplementary material for: Culture Dependent and Independent Analysis of Potential Probiotic Bacterial Genera and Species Present in the Phyllosphere of Raw Eaten Produce
Source: Int J Mol Sci. 2019 Jul 26;20(15):3661. doi: 10.3390/ijms20153661 (PMC6696213; doi:10.3390/ijms20153661)
Supplement: Supplementary file 1 [file ijms-20-03661-s001.zip › supplementary files/Supplemental Figure 1_final_SP.docx]

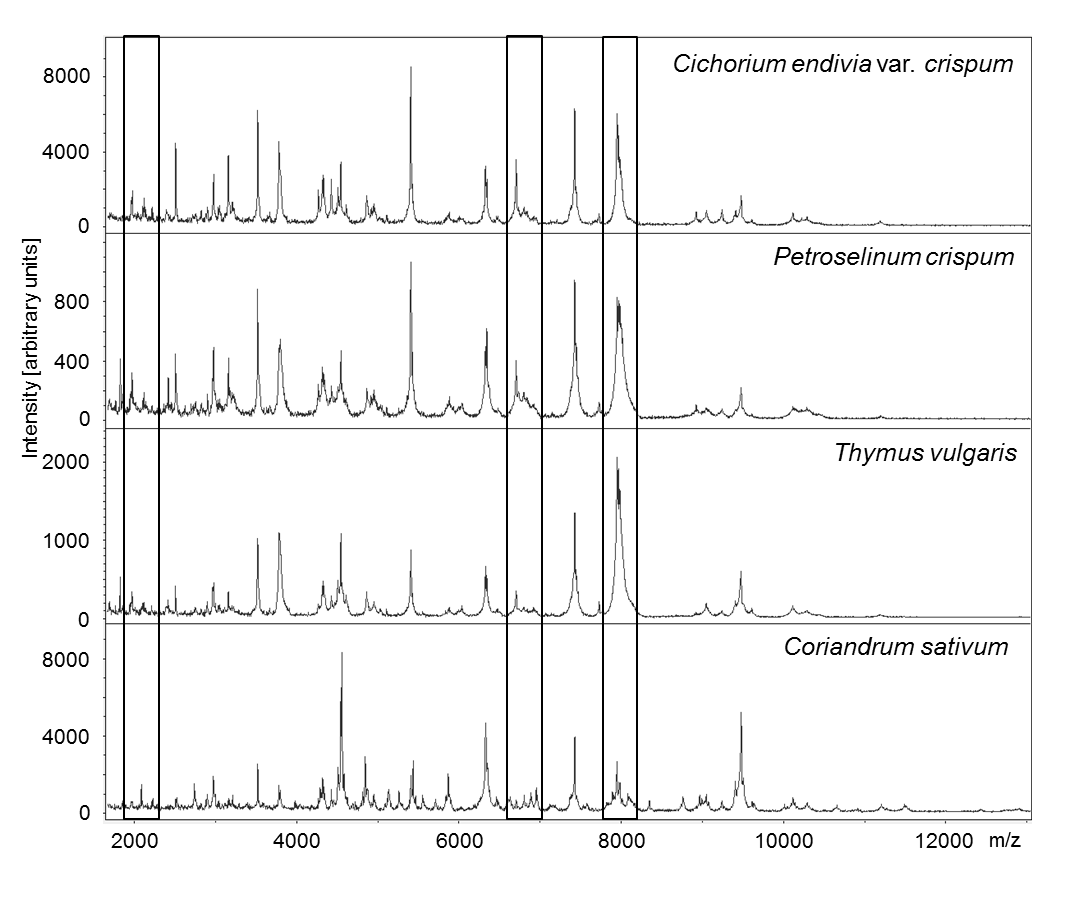


Supplemental Figure 1: Exemplary MALDI-TOF MS profiles obtained from strains that all were identified as *Pediococcus pentosaceus*. The strains were isolated from the phyllosphere of four different plant species, as shown in the figure. Some strain-specific peak pattern are indicated by boxes.
