# Supplementary material for: Culture Dependent and Independent Analysis of Potential Probiotic Bacterial Genera and Species Present in the Phyllosphere of Raw Eaten Produce
Source: Int J Mol Sci. 2019 Jul 26;20(15):3661. doi: 10.3390/ijms20153661 (PMC6696213; doi:10.3390/ijms20153661)
Supplement: Supplementary file 1 [file ijms-20-03661-s001.zip › supplementary files/Supplemental Figure 2_final_SP.docx]

Supplemental Figure 2: Heatmap of genus absence-presence patterns (respectively white and black) shown for the microbiome of *Lepidium sativum* phyllosphere of two independent experiments (Repl_1, Repl_2) comprising 4 replicates each. Genus specific OTUs were clustered regarding their binary absence-presence pattern, using the binary method of the R dist() function, whereas the samples were grouped based on the UPGMA clustering algorithm.
